# Adenoid cystic carcinoma of the salivary glands: a pilot study of potential therapeutic targets and characterization of the immunological tumor environment and angiogenesis

**DOI:** 10.1007/s00405-023-07884-3

**Published:** 2023-03-01

**Authors:** Ioannis Michaelides, Julian Künzel, Tobias Ettl, Philipp Beckhove, Christopher Bohr, Christoph Brochhausen, Andreas Mamilos

**Affiliations:** 1 Department of Otorhinolaryngology, Head and Neck Surgery, University Hospital Regensburg, Franz-Josef-Strauß-Allee 11, 93053 Regensburg, Germany; 2 Department of Oral and Maxillofacial Surgery, University Hospital Regensburg, Regensburg, Germany; 3 Division of Interventional Immunology, RCI Regensburg Center for Interventional Immunology, Franz-Josef-Strauß-Allee 11, 93053 Regensburg, Germany; 4 Institute of Pathology, University of Regensburg, Franz-Josef-Strauß-Allee 11, 93053 Regensburg, Germany; 5 Institute of Pathology, University Medical Centre Mannheim, Mannheim, Germany

**Keywords:** Adenoid cystic carcinoma, Macrophage polarization, Neovascularization, Salivary gland cancer

## Abstract

**Background:**

Adenoid cystic carcinoma (ACC) is a rare type of cancer commonly occurring in salivary glands. It is characterized by slow but infiltrative growth, nerve infiltration and overall poor prognosis, with late recurrence and distant metastasis. The treatment of ACC is still limited to surgery and/or (adjuvant) radiotherapy. Till now no promising systemic therapy option exists. However, various studies deliver promising results after treatment with anti-angiogenetic agents, such as anti-EGFR-antibody Cetuximab or Tyrosinkinase inhibitor Lenvatinib.

**Methods:**

By using of immunohistological methods we analyzed and compared the macrophage and lymphocyte populations, vascularization, and PD-L1-status in 12 ACC of the salivary glands.

**Results:**

All cases showed a significant elevation of macrophages with M2 polarization and a higher vascularization in ACC compared to normal salivary gland tissue. The CD4/CD8 quotient was heterogenous. ACC does not show relevant PD-L1 expression.

**Conclusions:**

The predominant M2 polarization of macrophages in ACC could be responsible for elevated vascularization, as already been proved in other cancer types, that M2 macrophages promote angiogenesis.

## Introduction

Salivary gland malignancies are overall rare and almost one fifth of them are adenoid cystic carcinomas (ACC) [1]. Based on its growing pattern, ACC is divided in three histological types, namely, cribriform, tubular and solid. In total the cribriform type has the best and solid type the worst prognosis [2]. An infiltration of nerves is also a typical feature of ACC and is correlated with a poorer outcome of the disease [3].

Former studies have shown an elevated expression of epidermal growth factor receptor (EGFR) in ACC [4, 5]. This suggests a high tendency to neovascularization, so that ACC might be susceptible to anti-angiogenetic agents, such as Cetuximab or Lenvatinib. On the other hand ACC show a weak to no programmed death ligand-1 (PD-L1) expression [6], a decisive biomarker for the use of Pembrolizumab in head and neck squamous cell carcinoma, which makes the benefit from immune checkpoint inhibitors questionable. ACC are low immunogenic tumors with low infiltration rates through CD8-positive lymphocytes [6], which is a crucial part of the anticancer immune response in the tumor microenvironment that can affect the outcome [7–9]. Furthermore, the CD4/CD8 ratio is in some cancer types, like the triple negative breast cancer, a useful prognostic tool [10], but can also be used as indicator to monitor the course of a therapy with immune checkpoint inhibitors [11]. However, its relevance in ACC is not yet analyzed.

Angiogenesis is a critical part of tumor growth and progression in general [12] and previous reports indirectly suggest elevated angiogenesis in ACC [13, 14], whereas a similar immmunohistological study did not show any significant difference between intratumoral and peritumoral vascularization [15]. Macrophages are a heterogenous group of cells that polarise to a M1 or M2 phenotype and are able to retain their plasticity and transform according to environmental signals [16–18]. M2-polarized macrophages, which have been linked to angiogenesis and cancer growth in pancreas [19], are also strongly represented in ACC [20, 21] and perhaps, at least partially, responsible for enhanced angiogenesis in this cancer. Till Dato no study to our knowledge compared both aspects.

In the era of personalized medicine, where monoclonal antibodies revolutionised oncology, the treatment of ACC does not appear to profit from these developments, as its curative therapy is still limited to surgery with/or radiation [22] and the therapy outcome is still not satisfactory. Up to now, no systemic treatment seems to be able to achieve satisfactory results [23–25].

In our study we used immunohistochemical methods to investigate the immunological microenvironment including vascularization of tumor and normal salivary gland tissue to examine if there are any abnormalities, that could be used as basis for translational development of new therapy regimens for ACC.

## Materials and methods

### Patients

**Fig. 1:**
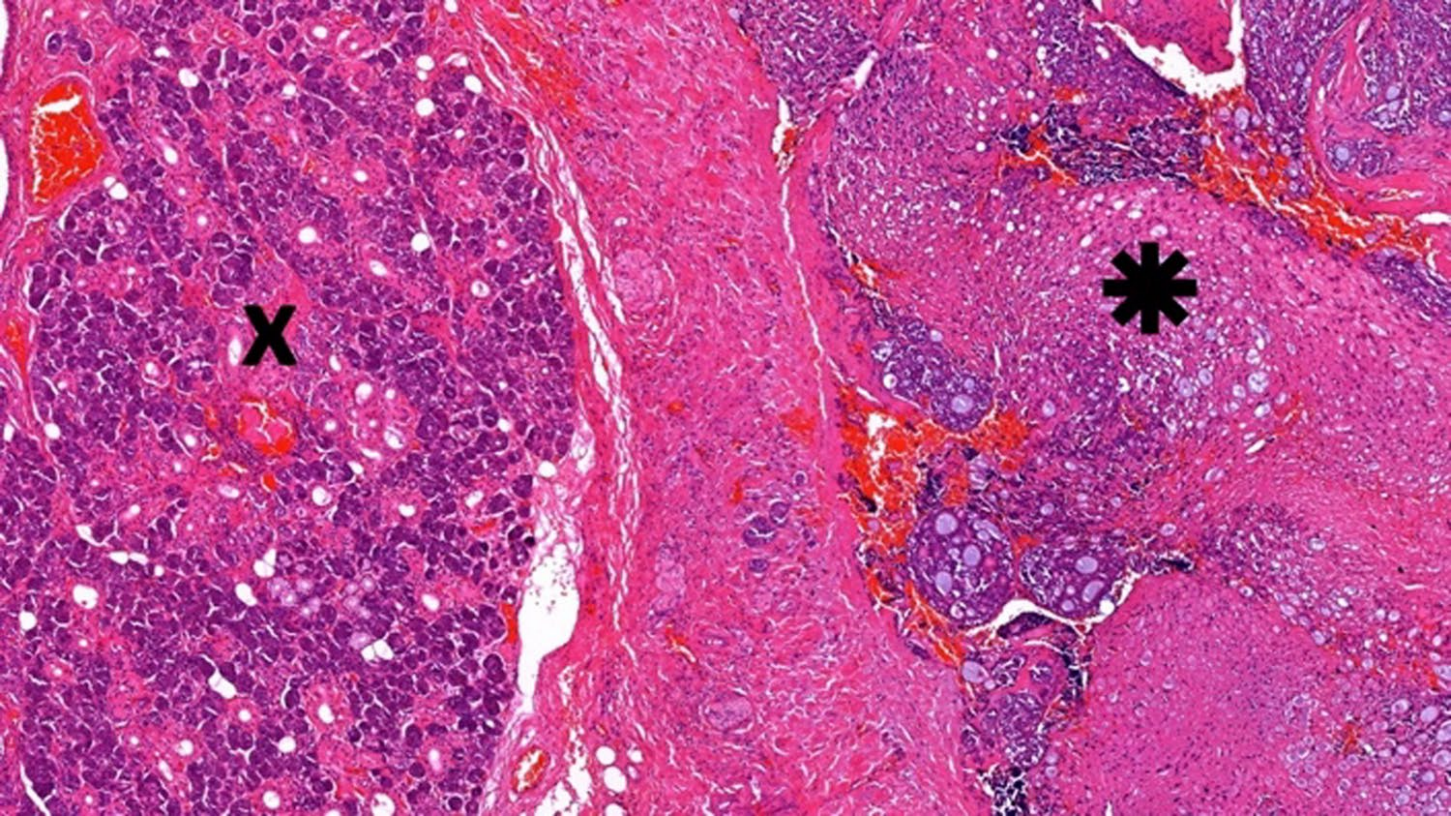
Overview of an evaluated case with salivary gland (X) and adenoid cystic carcinoma (✱). H.E.-Stain

Twelve Patients (8 female, 4 male) with mean age at time of diagnosis of 59.5 years, treated at the University Hospital of Regensburg, Germany were retrospectively included in this study (Table 1). The diagnosis of ACC was reviewed and confirmed. Tissue from the pathological routine diagnostics from these 12 primary ACC of the salivary glands was used for examination. After achieving a positive ethics votum (22-2753-104) an in situ immunohistochemical characterization of the immunological tumor environment was performed. For the in-situ characterization, standard routine diagnostic procedures and antibodies were applied. First, a new hematoxylin–eosin slide was prepared (Fig. 1).

**Table 1:** Patient characterization, tumor location and stadium, as PD-L1 Expression

Sex	Age	Localisation	UICC 8th Ed	TNM 8thEd	Pn	CPS	IC (%)	TPS (%)
W	46	Right submandibular gland	IVA	pT4a,cN0,cM0	Pn1	< 1	< 1	0
W	39	Right parotid gland	III	pT3,cN0,cM0	Pn1	0	0	0
W	87	Left sublingual gland	IVA	pT4a,cN0,cM0	Pn1	3	2	0
W	45	Left submandibular gland	II	pT2,pN0,cM0	Pn1	0	0	0
W	39	Hard palate	I	pT1,cN0,cM0	Pn0	0	0	0
M	69	Right parotid gland	IVA	pT4a,pN0,cM0	Pn1	0	0	0
W	45	Left parotid gland	I	pT1,pN0,cM0	Pn0	0	0	0
W	72	Left parotid gland	IVA	pT3,pN2b,cM0	Pn1	0	0	0
M	70	Right parotid gland	IVA	pT3,pN2b,cM0	Pn1	0	0	0
M	36	Left submandibular gland	I	pT1,pN0,cM0	Pn1	0	0	0
W	88	Left parotid gland	IVA	pT4a,cN0,cM0	Pn1	0	0	0
M	73, 67	Left parotid gland	I	pT1,pN0,cM0	Pn0	0	0	0

*Pn* Perineural-invasion, *UICC* Union for International Cancer Control

The tumor and residual salivary gland tissue were identified. In three tissue samples no normal salivary gland tissue was available for examination. Then, immunohistochemical reactions for PD-L1 (Dako Anti-Human PD-L1 Clone 22C3), CD4 (Ventana anti-CD4 Rabbit Monoclonal Primary Antibody Clone SP35) and CD8 (Ventana anti-CD8 Rabbit Monoclonal Primary Antibody, Clone SP57) for lymphocytes, CD68 (Dako Anti-Human CD68, Clone KP1) for macrophages and CD163 (NovocastraTM Lyophilized Mouse Monoclonal Antibody CD163, Clone 10D6) for M2-macrophages were performed. In addition, a CD31 (Dako Monoclonal Mouse Anti-Human CD31, Clone JC70) reaction was used to identify the vessel endothelial cells. All immunohistochemical stains were performed on tissue sections (2–4 µm thickness), prepared from-formalin-fixed (4% neutral buffered formalin) paraffin-embedded tissue blocks. Immunohistochemical staining was performed using a Roche Ventana Benchmark Ultra automated slide stainer (Ventana Medical Systems, Roche, France).

**Fig. 2:**
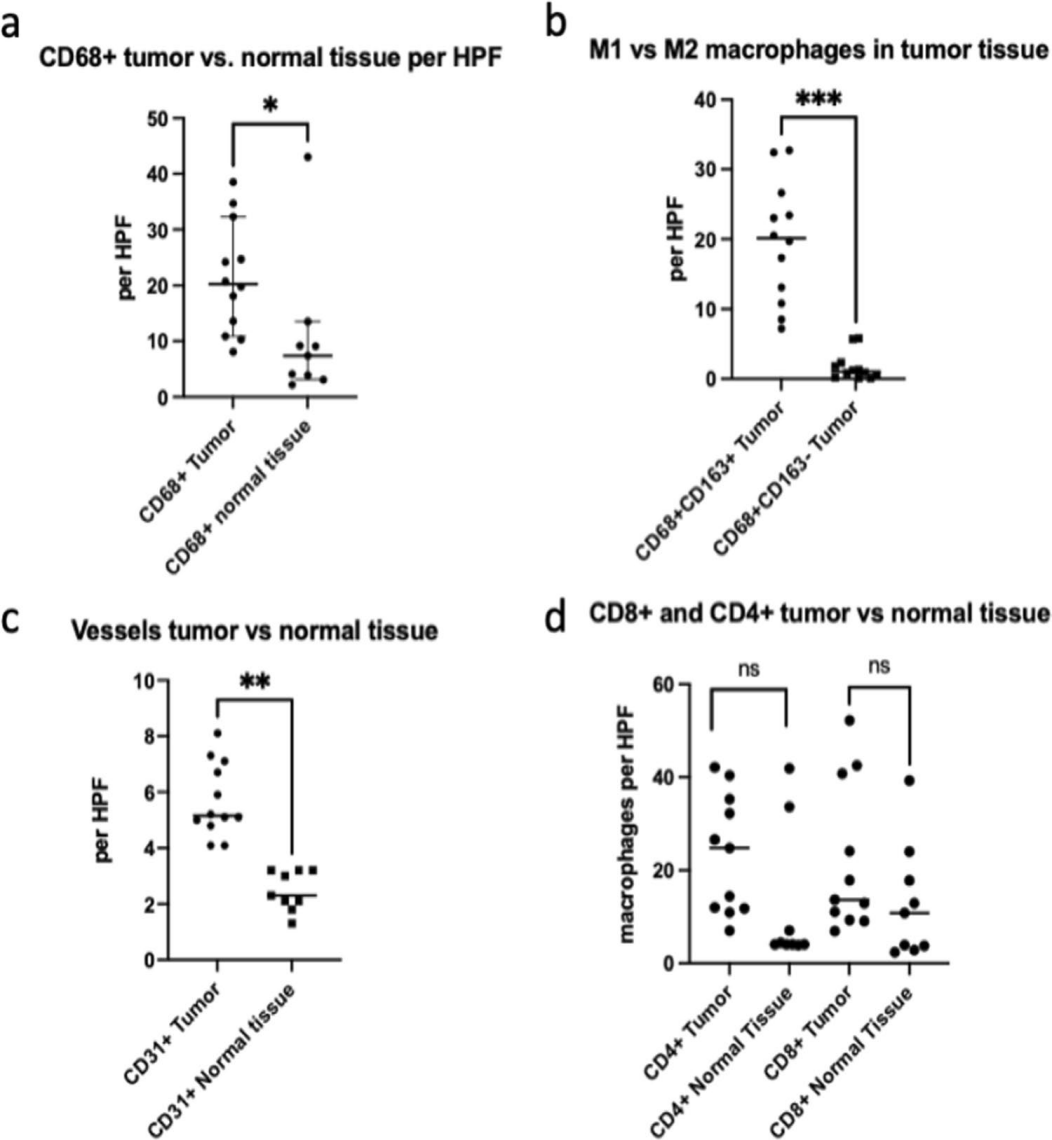
Comparison of the vascularization and immune cells in ACC and normal salivary gland tissue. Dots representing the mean number of counted cells or vessels per high power field (HPF). **a** CD68 + CD163− macrophages (M1) versus CD68 + CD163 + macrophages (M2) only in tumor tissue. **b** CD68 + macrophages, **c** CD31 + vessels and **d** CD8 + and CD4 + lymphocytes in tumor and normal salivary gland tissue. *p* < 0.05; **p < 0.005; ***p < 0.001

**Fig. 3:**
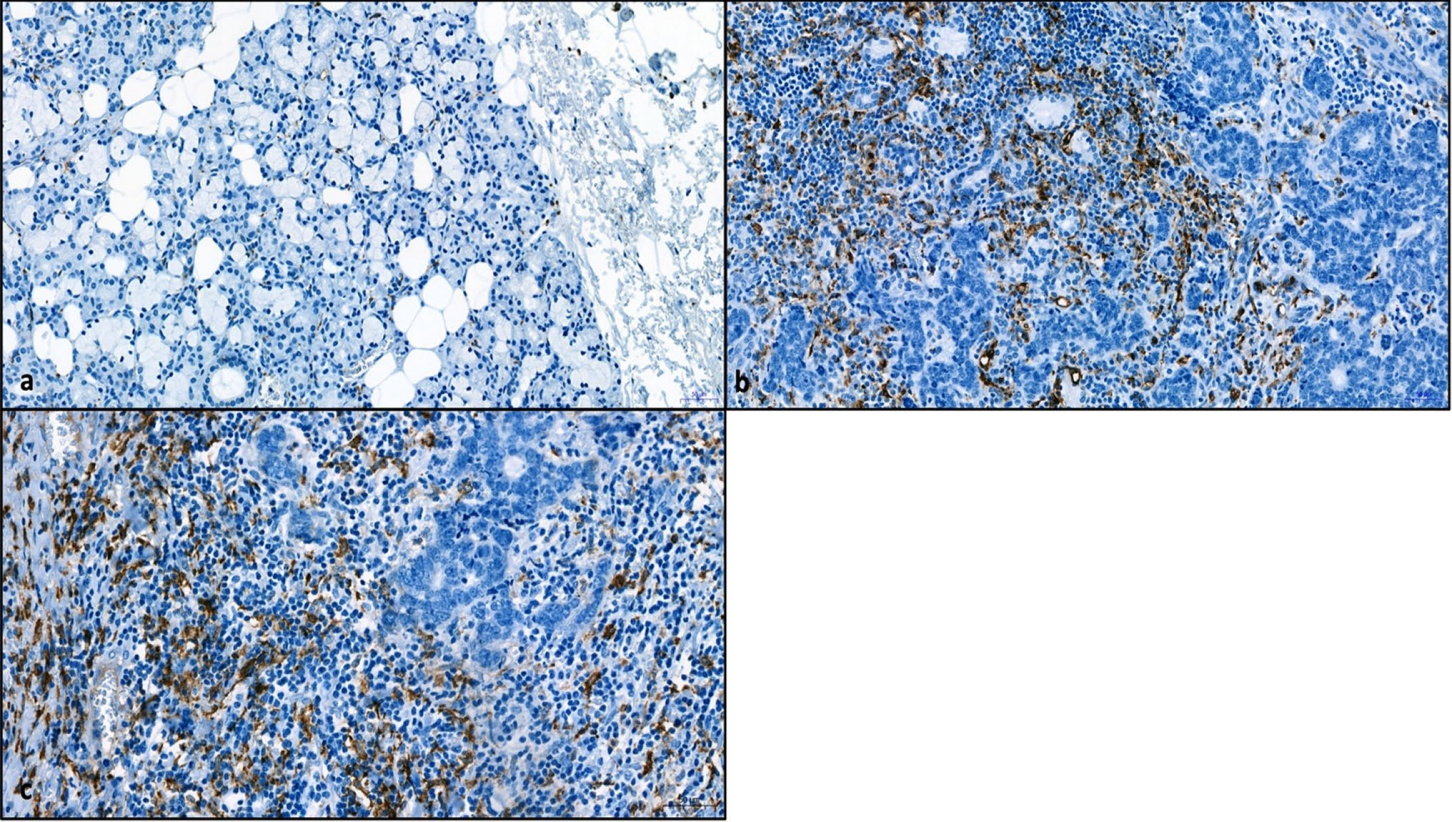
Immunohistochemical comparison of CD68 + Macrophages in salivary gland **a** ACC, **b** and CD163 + Macrophages in ACC (**c**). Immunohistochemical reaction for CD68 and CD163 in brown

All slides were scanned (3DHISTECH Ltd.Pannoramic slide scanner 250) and evaluated using a virtual microscopy software (3DHISTECH Ltd. Case Viewer Ver.2.2) (Figs. 2,  3).

For PD-L1 the three common diagnostic scores [tumor proportion score (TPS), Immune Cell Score (IC) and combined positive Score (CPS)] were assessed according the guidelines by an experienced consultant pathologist [26].

To quantify the lymphocytes, ten high power fields (HPF) of the invasion tumor front were examined and the positive cells were counted. The averages and then the quotient of CD4 to CD8 lymphocytes were calculated.

The CD68 reaction was used to visualize all macrophages. To evaluate the quantity of the macrophages ten HPF of the invasion tumor front and normal salivary gland tissue were examined and the positive cells were counted. Then, the mean value (Confidence interval 95%, CI 95%) of macrophages within the tumor front and the salivary gland tissue was calculated. The macrophages were then further characterized concerning their subpopulations. M2-macrophages were detected using an anti-CD163-antibody. Utilising the same, previously described procedure for the quantification of CD68-positive macrophages, the M2-macrophages were also quantified. Using the numbers of CD68-positive-macrophages and CD163-positive–M2-macrophages the number of M1-macrophages per HPF was calculated.

For the quantification of blood vessels, the endothelial cells were marked using a CD31-reaction. The vessel count in cross sections was performed within the tumour and normal salivary gland tissue, examining the same ten HPF, respectively, followed by calculation of the mean values like previously described.

### Statistical analysis

All acquired results were documented in Microsoft Excel 16.58 (Microsoft Corporation, Redmond, WA, U.S.A.). The calculations and graphs were also produced with GraphPad Prism version 9.3.1 for MacOS (GraphPad Software, San Diego, California USA). To investigate if there are significant differences between M1 and M2 macrophages, but also vessels and CD4 + and CD8 + lymphocytes in tumor and healthy salivary gland tissue, we applied the Wilcoxon signed ranged test. A *p* value ≤ 0.05 in two tailed tests was considered as statistically significant.

## Results

### The majority of macrophages in ACC shows an M2 polarization

In eight samples statistically significant more macrophages were observed in the tumor front in comparison with normal salivary gland tissue (Fig. 2a). In one sample nearly the same number of macrophages was found within the salivary gland tissue and the tumor, whereby this sample also showed signs of sialadenitis. For the three remaining samples a comparison of macrophages within the tumor and salivary gland was not possible due to lack of tumor free salivary gland tissue. Despite this, the macrophages of the tumor were calculated in all cases and further characterized in subpopulations of M1 and M2 macrophages. Interestingly, the majority of macrophages was polarized to an M2-phenotype (*p* = 0.0005), whereas only a small number of macrophages represented the M1-phenotype (Figs. 2b and  3).

### Elevated intratumoral vascularization compared to normal salivary gland tissue

**Fig. 4:**
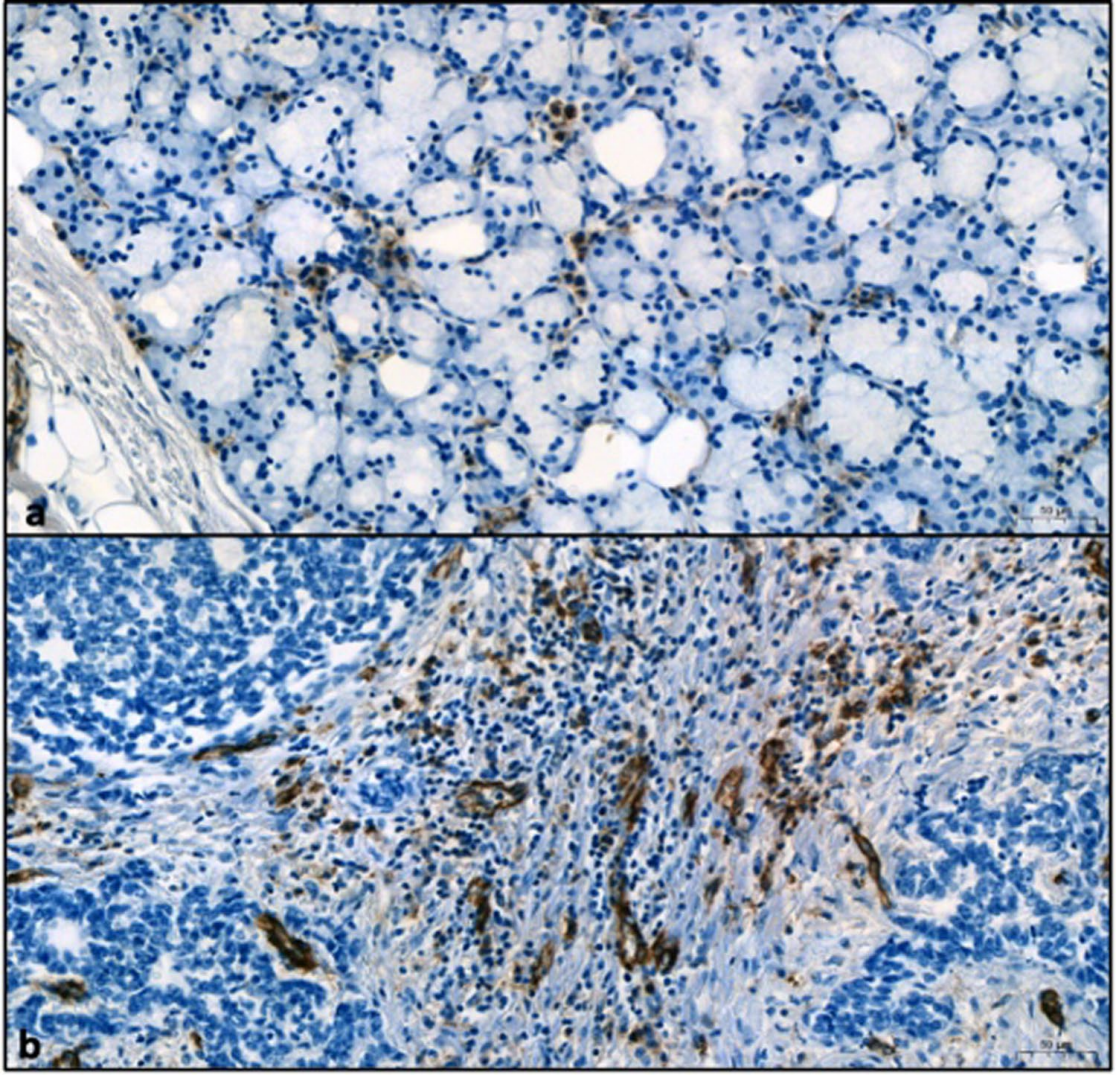
Immunohistochemical comparison of CD31-positive vessels in salivary gland (**a**) and ACC (**b**). Immunohistochemical reaction for CD31 in brown

In the analyzed tissues, in which both tumor and normal salivary gland tissue were available, we observed a much higher vascularization in the tumor compared to normal tissue. Intratumoral vessels were in average elevated by 2.89 times in the tumor tissue compared to local salivary gland tissue (*p* = 0.0039) (Figs. 2c and  4).

### Heterogeneous lymphocytes infiltration in ACC

Besides three cases, where no normal salivary gland tissue was available and another one in which no more tissue was available for examination, the intratumoral CD4 positive lymphocytes where elevated from 1.7 to 9.9 times compared to normal tissue, but the presence of CD4-positive lymphocytes was not in the same range. In six cases the range of intratumoral CD8-positive lymphocytes was elevated up to 17 times. In general though, comparing the number of lymphocytes in the tumor and normal tissue, revealed no statistically significant difference (Fig. 2d). After evaluating and comparing the quotient of CD4-to-CD8 intratumoral lymphocytes, we observed heterogenous results. In four out of eleven cases more CD4 lymphocytes were found and in seven cases the CD8 lymphocytes compared to CD4 lymphocytes dominated.

### PD-1 status in ACC

In terms of PD-L1 status no relevant expression was observed. More specifically all cases showed a TPS of 0. The vast majority of the samples (10 out of 12) also showed an IC and CPS of 0. In two cases we observed slightly positive IC and CPS scores with a maximum of 2% and 3%, respectively (Table 1). This shows that the tumor cells show no immuno-histochemical expression of PD-L1. In addition, the immunal cells show no or minimal positivity for the immunohistochemical reaction for PD-L1.

## Discussion

In the present study we clearly demonstrated an elevated number of macrophages in the tumor environment in comparison with normal salivary gland tissue but even more interesting is the finding of the almost complete polarization of macrophages in CD163-positive–M2-macrophages. It is well-known that the polarization of macrophages affects in various ways the behavior of tumors [27, 28]. M2 macrophages promote tumor aggression and progress [19, 29] in contrary to M1-macrophages which have an antitumoral effect [28]. They are also known for their role in the tumor angiogenesis [19, 30]. This is also consistent with our findings, showing significantly more vessels within the tumor tissue compared to normal salivary gland tissue (*p* = 0.0039), as well as significantly more M2 macrophages in comparison with M1 macrophages.

Recently Yang et al. showed in their experiments, that in ACC the mechanism of CCL2/CCR2 axis in the interactions between tumor cells and tumor-associated macrophages during the progression of salivary ACC, promotes a polarization to M2 tumor-associated macrophages [20], which explains the significantly higher polarization (*p* = 0.0005) of macrophages in the M2-subpopulation found in our study.

Adenoid cystic carcinomas are rare tumors and represent a challenge for clinicians. Confirming already performed studies, ACC are negative or extremely low positive for PD-L1 (all three scores), also suggesting a failure of immune check point inhibitor therapy.

The CD4/CD8-ratio of tumor infiltrating lymphocytes possesses in some malignancies—like gastric cancer [31] or as previously mentioned in the triple negative breast carcinoma [10]—a predictive value. In our study the ACC shows great heterogeneity in this matter and of course a larger collective is necessary to proof its clinical value.

These results, especially concerning the elevated macrophage infiltrate of the tumor front, their obvious polarization in M2 Macrophages and the also significantly elevated neovascularization, could help to develop new strategies of treating ACC. Potential inhibition of the angiogenesis, using well-known anti-angiogenetic agents may bring an additional effect regarding the cure of ACC. Our results support various studies and reports involving Cetuximab in the treatment of ACC showing promising results [32–36]. In addition, the tyrosine kinase inhibitor (TKI) Lenvatinib achieved good results in the treatment of patients with recurrent or metastasized ACC [37, 38], as well as other TKI-like sorafenib [39, 40] and axitinib [41]. However, there is a lack of prospective randomized controlled phase III studies that confirm the effectiveness of such therapies, mainly because of the rarity and slow growth of these tumors. Furthermore, controlling the population of M2-macrophages in tumor is already being investigated as a potential cancer treatment with promising results, but this concept is still far from finding its way to routine daily practice [42]. Nevertheless, targeting tumor-associated macrophages might be a breakthrough in the treatment of ACC, especially in patients with metastatic or advanced disease, which lack options of effective therapy. As soon as such substances are available, they should be investigated in animal models and also phase II and III studies to find their way in the clinical practice if they prove to be effective. It is, therefore, necessary that large cancer centers cooperate and perform together such studies to collect the essential number of patients, so that highly qualitative and representative results can lead to novel therapies for ACC. ACC is a complicated deadly systemic disease so a wider therapeutical approach, targeting various features of the tumor itself or its environment is in our opinion the key to treat this tumor entity or at least control its course.

In the study of Li et al. showed recently that macrophage migration inhibitory factor was overexpressed in ACC. MIF might be a risk factor for ACC patients [43] and seems also to promote the perineural invasion in ACC patients [44]. Until now there are no clear data, how this factor affects the polarization of macrophages. This is a key point for examining in further studies.

Next step is to examine the general effect of M2-macrophages in ACC using molecular pathology techniques, where protein and RNA-based studies shall be performed.

We are aware that the small number of examined cases poses as a limitation of our study. However, all cases showed statistically consistent results, so that the power of the results is credible and confirmed. It is, therefore, necessary to perform larger studies to confirm our results and taking in consideration the rarity of ACC this would be easier with the cooperation of other large cancer centers.

## Conclusion

The fact that the ACC invasion front is being dominated by a large number of macrophages with M2 phenotype and also the highly elevated angiogenesis in the tumor tissue maybe the clue to treat a stubborn until today disease. Larger studies that require cooperation of many cancer centers are urgently required and we are willing to be a part of such national or international initiative.

## Data Availability

The data sets presented in this article are not freely available because of patient confidentiality and participant privacy terms.

## References

[1] Hellquist H, Skalova A (2014). Adenoid cystic carcinoma.

[2] Du F, Zhou CX, Gao Y (2016). Myoepithelial differentiation in cribriform, tubular and solid pattern of adenoid cystic carcinoma: a potential involvement in histological grading and prognosis. Ann Diagn Pathol.

[3] Liu X, Yang X, Zhan C, Zhang Y, Hou J, Yin X (2020). Perineural invasion in adenoid cystic carcinoma of the salivary glands: where we are and where we need to go. Front Oncol.

[4] Bell D, Roberts D, Kies M, Rao P, Weber RS, El-Naggar AK (2010). Cell type-dependent biomarker expression in adenoid cystic carcinoma. Cancer.

[5] Nightingale J, Lum B, Ladwa R, Simpson F, Panizza B (2021). Adenoid cystic carcinoma: a review of clinical features, treatment targets and advances in improving the immune response to monoclonal antibody therapy. Biochim Biophys Acta Rev Cancer.

[6] Mosconi C, de Arruda JAA, de Farias ACR, Oliveira GAQ, de Paula HM, Fonseca FP (2019). Immune microenvironment and evasion mechanisms in adenoid cystic carcinomas of salivary glands. Oral Oncol.

[7] Afonina IS, Cullen SP, Martin SJ (2010). Cytotoxic and non-cytotoxic roles of the CTL/NK protease granzyme B. Immunol Rev.

[8] Wang J, Li R, Cao Y, Gu Y, Fang H, Fei Y (2021). Intratumoral CXCR5+CD8+T associates with favorable clinical outcomes and immunogenic contexture in gastric cancer. Nat Commun.

[9] Oshi M, Asaoka M, Tokumaru Y, Yan L, Matsuyama R, Ishikawa T (2020). CD8 T cell score as a prognostic biomarker for triple negative breast cancer. Int J Mol Sci.

[10] Wang K, Shen T, Siegal GP, Wei S (2017). The CD4/CD8 ratio of tumor-infiltrating lymphocytes at the tumor-host interface has prognostic value in triple-negative breast cancer. Hum Pathol.

[11] Shindo G, Endo T, Onda M, Goto S, Miyamoto Y, Kaneko T (2013). Is the CD4/CD8 ratio an effective indicator for clinical estimation of adoptive immunotherapy for cancer treatment?. J Cancer Ther.

[12] Zuazo-Gaztelu I, Casanovas O (2018). Unraveling the role of angiogenesis in cancer ecosystems. Front Oncol.

[13] House SL, Castro AM, Lupu TS, Weinheimer C, Smith C, Kovacs A (2016). Endothelial fibroblast growth factor receptor signaling is required for vascular remodeling following cardiac ischemia-reperfusion injury. Am J Physiol Heart Circ Physiol.

[14] Myoken Y, Myoken Y, Okamoto T, Sato JD, Kan M, Mckeehan WL (1996). Immunohistochemical study of overexpression of fibroblast growth factor-1 (fgf-1), fgf-2, and fgf receptor-1 in human malignant salivary gland tumours. J Pathol.

[15] KoochekDezfuli M, Seyedmajidi M, Nafarzadeh S, Yazdani F, Bijani A (2019). Angiogenesis and lymphangiogenesis in salivary gland adenoid cystic carcinoma and mucoepidermoid carcinoma. Asian Pacific J Cancer Prev..

[16] Stout RD, Jiang C, Matta B, Tietzel I, Watkins SK, Suttles J (2005). Macrophages sequentially change their functional phenotype in response to changes in microenvironmental influences. J Immunol.

[17] Stout RD, Suttles J (2004). Functional plasticity of macrophages: reversible adaptation to changing microenvironments. J Leukoc Biol.

[18] Mosser DM, Edwards JP (2008). Exploring the full spectrum of macrophage activation. Nat Rev Immunol.

[19] Yang Y, Guo Z, Chen W, Wang X, Cao M, Han X (2021). M2 macrophage-derived exosomes promote angiogenesis and growth of pancreatic ductal adenocarcinoma by targeting E2F2. Mol Ther.

[20] Yang Z, Li H, Wang W, Zhang J, Jia S, Wang J (2019). CCL2/CCR2 axis promotes the progression of salivary adenoid cystic carcinoma via recruiting and reprogramming the tumor-associated macrophages. Front Oncol.

[21] Linxweiler M, Kuo F, Katabi N, Lee M, Nadeem Z, Dalin MG (2020). The immune microenvironment and neoantigen landscape of aggressive salivary gland carcinomas differ by subtype. Clin Cancer Res.

[22] Rodriguez-Russo CA, Junn JC, Yom SS, Bakst RL (2021). Radiation therapy for adenoid cystic carcinoma of the head and neck. Cancers.

[23] Mahmood U, Bang A, Chen YH, Mak RH, Lorch JH, Hanna GJ (2021). A randomized phase 2 study of pembrolizumab with or without radiation in patients with recurrent or metastatic adenoid cystic carcinoma. Int J Radiat Oncol Biol Phys.

[24] Papaspyrou G, Hoch S, Rinaldo A, Rodrigo JP, Takes RP, van Herpen C (2011). Chemotherapy and targeted therapy in adenoid cystic carcinoma of the head and neck: a review. Head Neck.

[25] Laurie SA, Ho AL, Fury MG, Sherman E, Pfister DG (2011). Systemic therapy in the management of metastatic or locally recurrent adenoid cystic carcinoma of the salivary glands: a systematic review. Lancet Oncol.

[26] Schildhaus HU (2018). Der prädiktive Wert der PD-L1-Diagnostik. Pathologe.

[27] Rhee I (2016). Diverse macrophages polarization in tumor microenvironment. Arch Pharmacal Res.

[28] Pan Y, Yu Y, Wang X, Zhang T (2020). Tumor-associated macrophages in tumor immunity. Front Immunol.

[29] Suarez-Lopez L, Sriram G, Kong YW, Morandell S, Merrick KA, Hernandez Y (2018). MK2 contributes to tumor progression by promoting M2 macrophage polarization and tumor angiogenesis. Proc Natl Acad Sci U S A.

[30] Jetten N, Verbruggen S, Gijbels MJ, Post MJ, De Winther MPJ, Donners MMPC (2014). Anti-inflammatory M2, but not pro-inflammatory M1 macrophages promote angiogenesis in vivo. Angiogenesis.

[31] Zurlo IV, Schino M, Strippoli A, Calegari MA, Cocomazzi A, Cassano A (2022). Predictive value of NLR, TILs (CD4+/CD8+) and PD-L1 expression for prognosis and response to preoperative chemotherapy in gastric cancer. Cancer Immunol Immunother.

[32] Caballero M, Sosa AE, Tagliapietra A, Grau JJ (2013). Metastatic adenoid cystic carcinoma of the salivary gland responding to cetuximab plus weekly paclitaxel after no response to weekly paclitaxel alone. Head Neck.

[33] Hitre E, Budai B, Takácsi-Nagy Z, Rubovszky G, Tóth E, Remenár É (2013). Cetuximab and platinum-based chemoradio- or chemotherapy of patients with epidermal growth factor receptor expressing adenoid cystic carcinoma: a phase II trial. Br J Cancer.

[34] Jensen AD, Krauss J, Weichert W, Debus J, Münter MW (2010). RadioImmunotherapy for adenoid cystic carcinoma: a single-institution series of combined treatment with cetuximab. Radiat Oncol.

[35] Hitre E, Budai B, Takacsi-Nagy Z, Rubovszky G, Toth E, Remenar E (2013). Cetuximab and platinum-based chemoradio- or chemotherapy of patients with epidermal growth factor receptor expressing adenoid cystic carcinoma: a phase II trial. Br J Cancer.

[36] Mueller SK, Haderlein M, Lettmaier S, Agaimy A, Haller F, Hecht M (2022). Targeted therapy, chemotherapy, immunotherapy and novel treatment options for different subtypes of salivary gland Cancer. J Clin Med.

[37] Tchekmedyian V, Sherman EJ, Dunn L, Tran C, Baxi S, Katabi N (2019). Phase II study of lenvatinib in patients with progressive, recurrent or metastatic adenoid cystic carcinoma. J Clin Oncol.

[38] Feeney L, Jain Y, Beasley M, Donnelly O, Kong A, Moleron R (2021). Centralised RECIST assessment and clinical outcomes with lenvatinib monotherapy in recurrent and metastatic adenoid cystic carcinoma. Cancers.

[39] Thomson DJ, Silva P, Denton K, Bonington S, Mak SK, Swindell R (2015). Phase II trial of sorafenib in advanced salivary adenoid cystic carcinoma of the head and neck. Head Neck.

[40] Locati LD, Perrone F, Cortelazzi B, Bergamini C, Bossi P, Civelli E (2016). A phase II study of sorafenib in recurrent and/or metastatic salivary gland carcinomas: translational analyses and clinical impact. Eur J Cancer.

[41] Locati LD, Cavalieri S, Bergamini C, Resteghini C, Alfieri S, Calareso G (2019). Phase II trial with axitinib in recurrent and/or metastatic salivary gland cancers of the upper aerodigestive tract. Head Neck.

[42] Hu G, Guo M, Xu J, Wu F, Fan J, Huang Q (2019). Nanoparticles targeting macrophages as potential clinical therapeutic agents against cancer and inflammation. Front Immunol.

[43] Li C, Chen Q, Tian Z, Li S, Gong Z, Lin Z (2019). Expression of MIF, Beclin1, and LC3 in human salivary gland adenoid cystic carcinoma and its prognostic value. Medicine (Baltimore).

[44] Zhang M, Li ZF, Wang HF, Wang SS, Yu XH, Wu JB (2019). MIF promotes perineural invasion through EMT in salivary adenoid cystic carcinoma. Mol Carcinog.

